# Micro- and Nano-assembly of Composite Particles by Electrostatic Adsorption

**DOI:** 10.1186/s11671-019-3129-1

**Published:** 2019-08-28

**Authors:** Wai Kian Tan, Yuichi Araki, Atsushi Yokoi, Go Kawamura, Atsunori Matsuda, Hiroyuki Muto

**Affiliations:** 1 Institute of Liberal Arts and Sciences, Toyohashi University of Technology, 1-1, Hibarigaoka, Tempaku-cho, Toyohashi, Aichi 441-8580 Japan; 2 Department of Electrical and Electronic Information Engineering, Toyohashi University of Technology, 1-1, Hibarigaoka, Tempaku-cho, Toyohashi, Aichi 441-8580 Japan

**Keywords:** Composite materials, Electrostatic adsorption method, Microparticles, Nanoparticles, Self-assembly

## Abstract

This work reports on a novel controlled nanocomposite fabrication technique which is applicable for material design via a micro- and nano-assembly method. The principle is based on the use of electrostatic adsorption of the surface charge-modified particles via layer-by-layer assembly. The polarity and the zeta potential of the surface charge was controlled using polycation and polyanion, while the zeta potential strength was controlled via the number of alternating coating layers which was determined using zeta potential measurement. A systematic study was conducted to demonstrate the feasibility of composite material assembly via electrostatic adsorption using alumina (Al_2_O_3_) and silica (SiO_2_) composite as a study model, which was carried out as a function of surface zeta potential, surface coverage percentage, and processing time. The considerable potential of this technique for composite material design is also further demonstrated with controlled assembly involving different materials in various structural forms such as fiber, whisker, nanosheets, and even irregular-shaped foam-like structured urethane. The composite materials designed using this EA method possess good potentials to be utilized for various applications such as mechanical property control, composite ceramic films formation, selective laser sintering, and rechargeable metal-air battery.

**Electronic supplementary material:**

The online version of this article (10.1186/s11671-019-3129-1) contains supplementary material, which is available to authorized users.

## Introduction

**Fig. 1:**
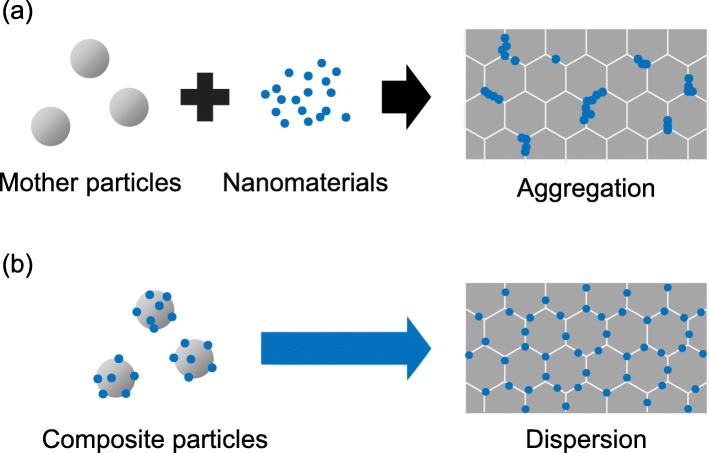
Comparison of the microstructures obtained using **a** conventional mechanical milling method and **b** novel electrostatic adsorption technique for the fabrication of composite materials

**Fig. 2:**
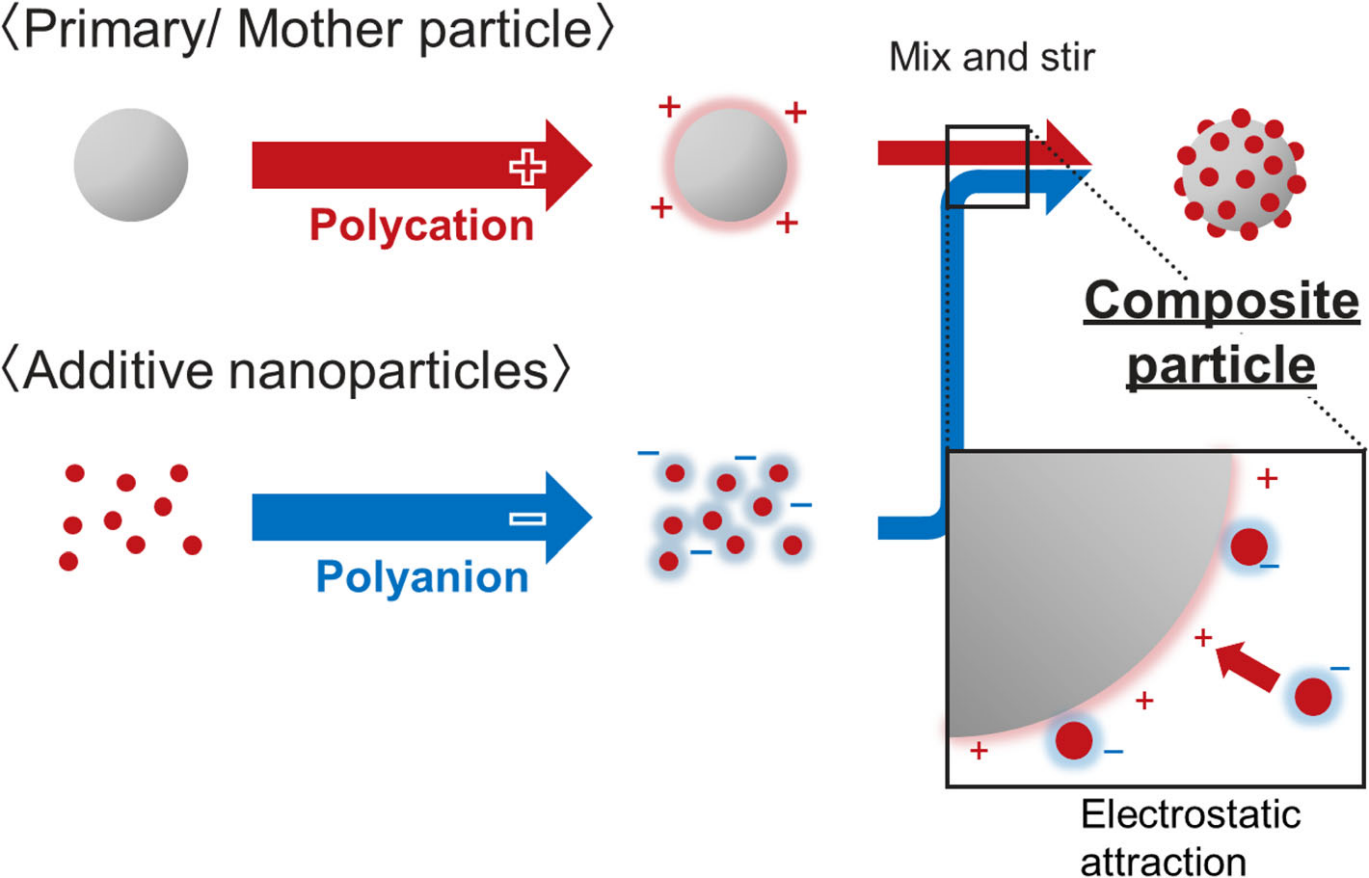
Schematic of surface electrostatic charge adjustment using polycation and polyanion for the formation composite particle

In the nanoscale-focused society nowadays, nano-architecture design and fabrication have never been more crucial and have seen rapid development in recent years. Bottom-up assemblies such as self-assembled monolayers and a layer-by-layer (LbL) assembly technique utilizing electronic adsorption have attracted significant interest from researchers [1–3]. This has led to a new concept known as nano-architectonics where an integration of hybrid polymer and inorganic nano-architecture is used for nanoscale morphological design [4]. Since the discovery by Decher et al., most of the reported work involved the formation of one or multiple layer of films (coatings) on the surfaces and focusing on surface molecular engineering [5, 6], conjugate polymers, bio-components, graphene, and fullerene [7]. LbL method has opened up significant potential for the development of advanced materials that require precise design such as core-shells for drug delivery and photonic crystals as well as selective functional molecules [8, 9]. However, controlled assembly of micro- and nanoparticles using an electrostatic adsorption technique is rarely reported [10]. Mo et al. demonstrated the assembly of ZnO nanorod-based hollow hemisphere assembly using hydrothermal thermolysis of Zn precursor in the presence of 2 g long chain polymer with a large side group, poly(sodium 4-styrenesulfonate) (PSS) [11]. They mentioned that the presence of a water-soluble long chain with a large side group is crucial for the formation of the unique assembled structure that consists of hollow hemispheres. The long chain is said to promote clustering of secondary colloidal particles that would lead to subsequent space-limited crystallization and dissolution. Using the similar concept, it is also demonstrated to be possible to fabricate three-dimensional polymer shells using cores that consisted of soluble colloidal template by Decher [12, 13] and Caruso et al. [14]. Their works have laid the foundation toward more material design using the EA method. It is also reported that the size and density of a material’s nanostructures applied during bio-engineering could specifically induce the desired biological properties [15, 16]. Visalakshan et al. have reported on a versatile and scalable controlled formation of covalently bonded Au particles on a plasma-deposited polymethlyoxazoline interlayer with a well-defined nano-topography that could be applied to biomaterial engineering-related technologies [15]. In another work reported by Li et al., they have demonstrated the feasibility to obtain bifunctional microspheres that consisted of Fe_2_O_3_ (core) and SiO_2_ (shell) with Au nanoparticles adsorbed on the surface through interaction with polyethyleneimine via LbL method. The bifunctional hybrid composite exhibited excellent catalytic performance in organic and inorganic reduction while possessing superparamagnetic property that enabled efficient separation using magnetic field [17]. The abovementioned works have further emphasized the importance of micro- and nano-assembly toward the generation of desired properties for various advanced functional applications. Looking forward toward large scale fabrication, Hueckel and Sacanna reported on a mix-and-melt reaction method that enables rapid mass production of anisotropic core-shell colloids using electrostatic self-assembly [18]. In the LbL method, despite the possible application of uncharged particles/colloids, the charged ones still remain the commonly used method through the assembly of multilayer polyelectrolyte [19]. Other than polyelectrolyte utilization, the zeta potential can also be controlled using pH adjustment depending on the material used in the composite formation [20, 21]. The sequential application of oppositely charged polyelectrolyte could increase the surface charge strength as well as stability of the polyelectrolyte coatings which can be determined using zeta potential measurement [13, 22]. When the zeta potential of a surface-charge modified particle is equivalent or more than +/− 40 mV, it is reported to be sufficient to obtain a good stable electrostatic interaction to avoid agglomeration and remain in colloidal form [21, 23]. Despite the advancement and development of the LbL method, its utilization for materials and composites design are rarely reported despite its huge potential. Therefore, in this work, we not only demonstrated a facile and more superior method to obtain a homogeneous materials mixture using the EA method but have also realized the feasibility of composite material design that crosses the boundaries between materials and shape. The potential of EA method was further expanded by demonstrating the feasibility of this method for decoration of desired additives onto irregular-structured materials such as urethane foam, sheet-like boron nitride (BN), and rod-like structured materials. As for conventional mixing methods such as mechanical milling, it is often the case where the structure of the precursor is either degenerated or altered due to volatile impact and heat generation. Moreover, agglomeration of the mixture also occurs which would then affect the final properties of the composite materials [21, 24, 25]. To overcome this issue, a method for precise nanoscale design of materials is indispensable to boost toward advanced precision manufacturing. Figure 1 is a schematic that shows the comparison of the microstructures that could be obtained via a conventional mechanical milling method with the aggregation occurrence compared to a novel homogenous decoration of composite materials via the EA method. A well-distributed microstructure could be obtained using the homogenously decorated composite materials to generate the desired properties from a functional composite material. In a recent reported work, a homogeneous decoration of indium tin oxide (ITO) nanoparticles on the surface of poly(methyl methacrylate) (PMMA) was demonstrated using the EA method. The ITO-PMMA composite powders obtained were then used to fabricate a pellet with good transparency in the visible-light region and a controllable IR light-shielding effect [21]. Therefore, a clear overview of the EA method for micro- and nano-assembly is vital to emphasize the feasibility and potential of this technique for material design. The fundamental principle for this novel work is illustrated in Fig. 2 where the control of surface charge is carried out using polyelectrolytes (polycation and polyanion) in order to enable homogenous decoration of desired additive particles on the primary/mother particle. By utilizing the attractive force, nanocomposites with significant homogeneity could be achieved regardless of structural complexity. The preservation of the primary and secondary nanostructures such as nanofibers and nanorods could be achieved compared to the conventional mechanical milling method which would destroy their original morphological structure. In terms of fabrication cost, the EA method is also a more cost-effective method. Freymann et al. also emphasized that the EA method is an excellent bottom-up assembly method for photonic crystals fabrication as opposed to the extremely expensive top-down approaches [8]. The demonstrated feasibility of the nano-architectural composite design demonstrated in this novel work could be a useful platform for various applications due to its cost-competitiveness and simplicity. Room temperature formation and the superior homogeneity of this method hold significant advantage for precise powder-based fabrication technology such as aerosol deposition [25], ceramic 3D printing technology, and additive manufacturing laser sintering[26, 27]. The applicability of this EA method toward useful practical applications is also demonstrated in our recently reported work for IR light shielding [21], mechanical property control of carbon-based alumina composite [24], and rechargeable Fe-air battery [20].

## Experimental Procedures

### Formation of Al_2_O_3_-SiO_2_ Composite Particles

**Fig. 3:**
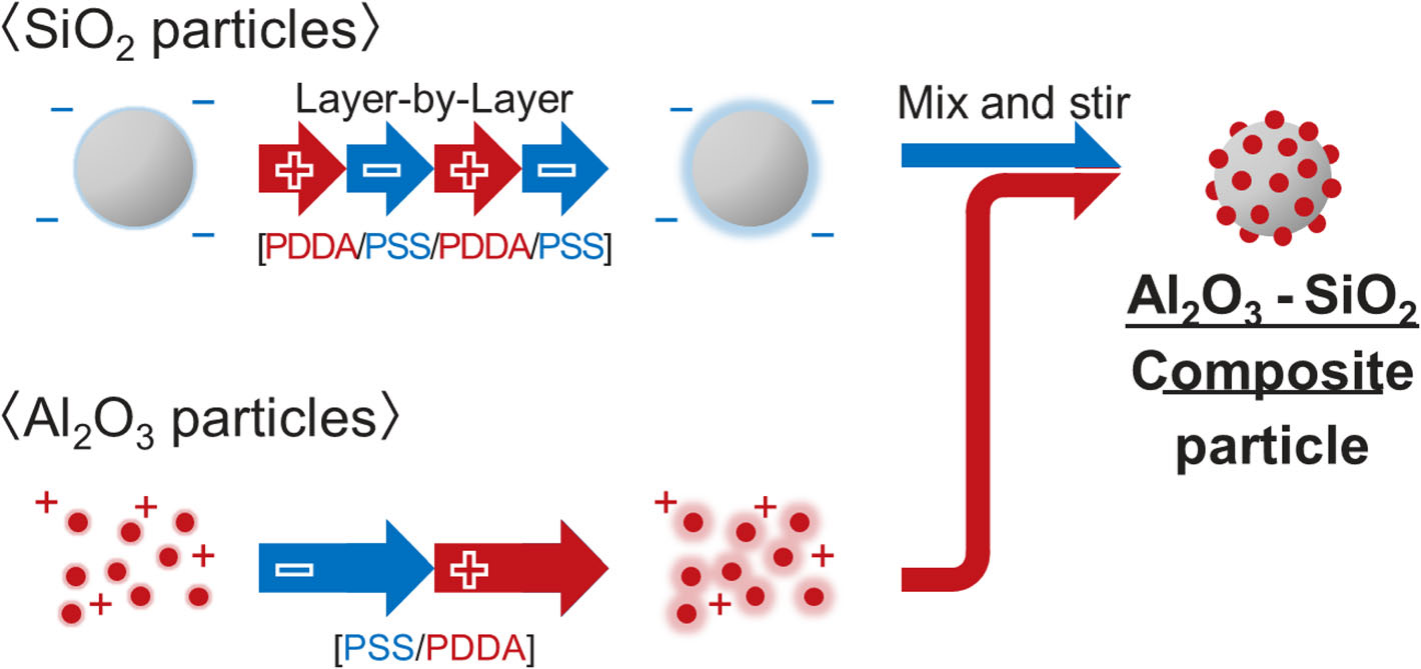
Schematic of a Al_2_O_3_-SiO_2_ composite particle formation after layer-by-layer coating process of PDDA and PSS

The experiments were carried out using commercially available monodispersed spherical SiO_2_ particles (average particle diameter 8.8 μm, Ube EXSYMO) and alumina (Al_2_O_3_) particles (average particle size 100 nm, Taimei Chemical Co., Ltd.). The polycation and polyanion used were polydiallyldimethylammonium chloride (PDDA) (average molecular weight 100,000 to 200,000, Sigma-Aldrich) and polysodium styrenesulfonate (PSS) (average molecular weight 70,000, Sigma-Aldrich), respectively. The primary SiO_2_ particles were immersed into the polyelectrolytes in the order of PDDA, PSS, PDDA, and PSS to induce a negative surface charge. As for the Al_2_O_3_ particles, the surface charge was prepared by immersion in PSS and PDDA in order to obtain a positive surface charge. The surface zeta potential was controlled by repetitive multiple-layer coating by LbL process [12, 13, 28]. Finally, the suspension of SiO_2_ and Al_2_O_3_ particles with the opposite zeta potential were mixed together. The pH of the solution prepared was within the vicinity of 7–8 (neutral). When PDDA or PSS was added into an aqueous solution, the pH of the solution changed to approximately 5.5 and 6.8, respectively. After the addition of PDDA and PSS, the solutions were then washed and rinsed several times (up to four times) to remove the excessive PDDA and PSS from the solution. After washing and rinsing, the pH returned to an original pH of approximately 7–8. Although the suspension obtained was stable for several days, they were used for the electrostatic assembly a few hours upon preparation. Smaller electrostatically charged particles (secondary) were attracted toward and attached onto the larger particles (primary) forming the nano/micro-composite particles. The schematic of the overall fabrication process of the Al_2_O_3_-SiO_2_ composite particles is shown in Fig. 3. The Al_2_O_3_ and SiO_2_ composite particles were mixed and stirred for up to 180 min to allow complete adsorption. In the investigation of coverage control, the amount of particle adsorption was adjusted by changing the mixing ratio of the precursors using the formula below:

1 $$ {W}_a=C{V}_a{\rho}_a\frac{W_m}{\rho_m{V}_m} $$

*W*_*a*_ is the added particle amount (g), *C* is the coverage ratio of the secondary particles on the primary particles (*C* = *S*_*a*_/*S*_*m*_; *S*_*a*_ is the cross-sectional area [m^2^] of the additive particles while *S*_*m*_ is the surface area of the primary particles [m^2^]), *V*_*a*_is the volume of one additive particle [m^3^], *ρ*_*a*_ is the density of additive particles [g/m^3^], *W*_*m*_ is the amount of the primary particles [g], *ρ*_*m*_ is the density of the primary particles [g/m^3^], and *V*_*m*_ is the volume of one primary particle [m^3^]. In this study, the amount of additive particles was adjusted to 25, 50, and 75% surface coverages of a constant amount of primary SiO_2_ particles. The coverage estimation of the Al_2_O_3_-SiO_2_ composite obtained was also calculated from the SEM images and tabulated in Table 1. In a different study on the effect of deposition time during the reaction process, the duration for Al_2_O_3_ and SiO_2_ composite particle formation was investigated at intervals of 5, 15, and 60 min with a constant coverage of 25%.

**Table 1:** The number of adsorbent particles and coverage estimated from SEM images

The targeted coverage (%)	The number of adsorbed particles (/μm^2^)	The estimated coverage (%)
25	29	22.8
50	62	48.7
75	95	74.6

### Formation of SiO_2_-SiO_2_ Composite Particles with Size Control

In this investigation, in order to demonstrate the feasibility of designing a composite that consists of the same material but of different sizes, SiO_2_ with particle sizes of 1, 4, and 16 μm (Ube EXYMO) were used. As for the primary 16-μm SiO_2_ particles, an LbL coating of PDDA/PSS/PDDA/PSS was carried out while as for the secondary smaller 1- and 4-μm SiO_2_ particles, LbL coating of PDDA/PSS/PDDA was performed. The suspensions were then mixed and stirred accordingly.

### Formation of Various Composite Combination Using EA Method

In order to further demonstrate the feasibility and flexibility of this novel method in composite design involving various materials and shapes, materials such as Al_2_O_3_, polymethyl methacrylate (PMMA), carbon nanotube (CNT), boron nitride (BN), carbon fiber, and silicon carbide (SiC) in the structural form of fiber, whisker, nanosheet, and an irregular foam-like structure were used to form the composites via the EA method. Depending on the material, the surface charge modification differs. For Al_2_O_3_ and SiO_2_ that possess positive and negative surface charge, respectively, PSS and PDDA were used to induce the opposite charge until the zeta potential is higher than +/− 40 mV prior to electrostatic assembly. For materials that exhibit low or negligible zeta potential such as PMMA, carbon microspheres, CNT, BN, carbon fiber, SiC, and urethane, an initial layer of surfactant, sodium deoxycholate (SDC), was used to coat and induce a negative surface charge on the surface followed by PDDA. Multiple alternating layers of PDDA and PSS were adjusted until the zeta potential is higher than +/− 40 mV prior to mixing to allow electrostatic assembly. For example, in order to obtain composite that consist of carbon-microsphere-Al_2_O_3_, the surface of primary particle Al_2_O_3_ was surface-charged modified using PSS to induce a negative surface charge. As for the secondary carbon microspheres, an initial coating of SDC was carried out and followed by PDDA in order to generate a positive zeta surface potential. If the zeta potential is less than + 40 mV, alternating coating of PSS/PDDA is carried out to obtain a higher and more stable surface potential for the electrostatic assembly. Then, the surface-charged modified Al_2_O_3_ and carbon microsphere aqueous solution were mixed and stirred in order to promote the electrostatic adsorption process. A similar approach was applied to PMMA, CNT, BN, carbon fiber, SiC, and urethane prior to electrostatic assembly process.

### Method, Morphological Observation, and Measurement

An ultrasonic homogenizer (QSonica, LLC., Q 700) was used to disperse the agglomerated particles in a solution. A freeze dryer (FDU-1200, Tokyo Science Instrument Co., Ltd.) was used for drying the composite particle suspension obtained. The morphologies obtained after EA were observed using an S-4800 Field Emission Scanning Electron Microscope (FE-SEM, Hitachi S-4800). The zeta potential was measured using measurement equipment from Otsuka Electronics Co. Ltd., ELSZ-1 and Micro Tech Nission, ZEECOM Co. Ltd.

## Results and Discussion

**Fig. 4:**
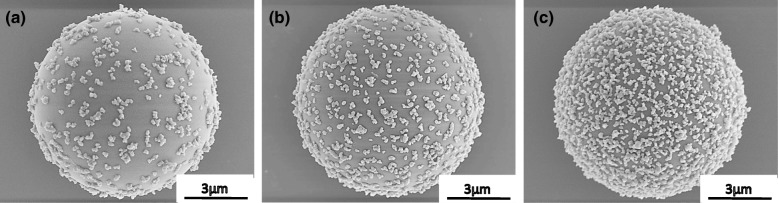
SEM images of the Al_2_O_3_-SiO_2_ composite particles with different coverage of **a** 25, **b** 50, and **c** 75%, respectively

**Fig. 5:**
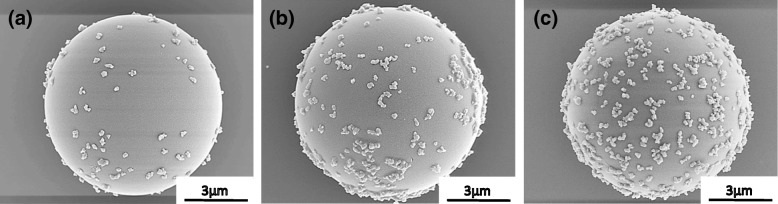
SEM images of the Al_2_O_3_-SiO_2_ composite particles that were mixed and stirred for **a** 5, **b** 15, and **c** 60 min, respectively, with a fixed additive particle amount of 25% coverage

**Fig. 6:**
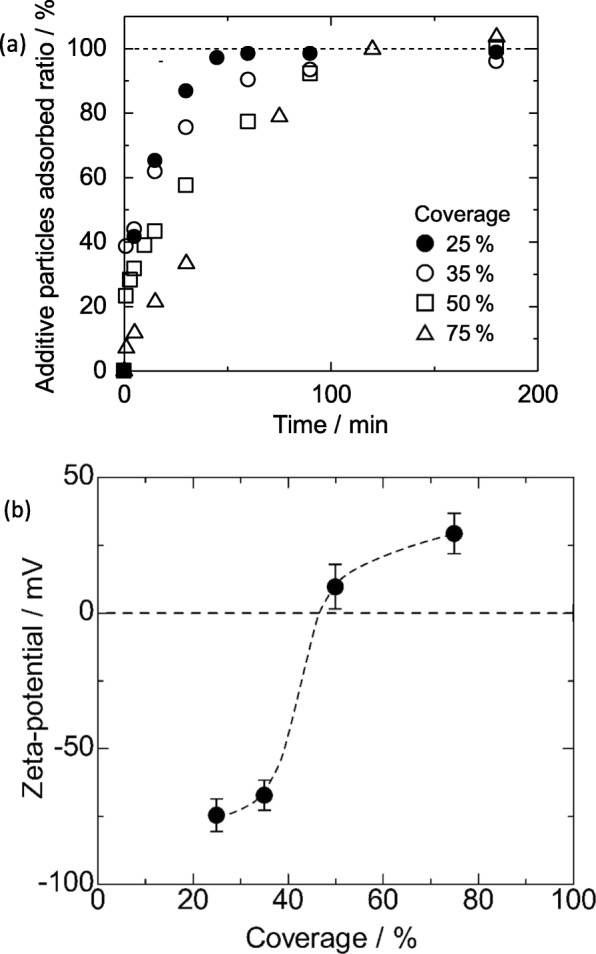
**a** Time dependence behavior of Al_2_O_3_ adsorption on SiO_2_ particle for various suspension coverage ratios. **b** Zeta potential of Al_2_O_3_-SiO_2_ composite particles obtained using varying coverage percentage. Three independent samples were prepared and an average of five measurements were carried out to obtain the standard deviation

Figure 4 shows the SEM images of the obtained Al_2_O_3_-SiO_2_ composite particles with the different coverages of 25, 50, and 75%, respectively. It can be clearly observed that the Al_2_O_3_ nanoparticles are distributed homogenously across the surface of the SiO_2_ particle. From the SEM images, the amount of Al_2_O_3_ particles adsorbed onto the surface of the SiO_2_ particles was calculated and is summarized in Table 1. From the results obtained, the values of estimated coverage measured are approximate to the intended target coverages of 25, 50, and 75%. This result shows that by using this novel EA method, the coverage could be controlled by adjusting the amount of additive particles while maintaining a very good homogenous coverage where the secondary Al_2_O_3_ particles are distributed evenly on the surface of primary SiO_2_ particle without any sign of agglomeration or concentrated patches. In the separate study of reaction time during the mixing and stirring, the SEM images of the composite particles (25% coverage) obtained after 5, 15, and 60 min are shown in Fig. 5. The amount of additive particles deposited on the primary SiO_2_ particle was observed to increase with time. It is noteworthy that even at a short mixing and stirring time of 5 min, the additive particles are seen to be distributed evenly on the surface of SiO_2_ particles (not agglomerated). With prolonged mixing and stirring times of 15 and 60 min, the amount of deposited Al_2_O_3_ increased accordingly. Upon 60 min, the amount of Al_2_O_3_ particles obtained on SiO_2_ is similar to that obtained in the abovementioned 25% distribution coverage. This shows that the particle deposition of the EA method is dependent on the reaction time (mixing and stirring). The overall time dependence behavior of Al_2_O_3_ adsorption on SiO_2_ for different coverage ratios as well as the zeta potential measured are summarized in Fig. 6. From Fig. 6a, in order to obtain coverage of 25%, the time required to achieve a deposition plateau is 60 min while those with higher surface coverage required a prolonged time up to 180 min. The deposition delay is caused by the increase of Al_2_O_3_ particle suspension leading to the increased Al_2_O_3_ adsorption on SiO_2_ that subsequently resulted in the occurrence of a steric hindrance effect [5, 29]. From Fig. 6b, it can be seen that the apparent zeta potential of Al_2_O_3_-SiO_2_ composite gradually shifted from negative to positive with increasing additive Al_2_O_3_ particle coverage. As the amount of positively charged Al_2_O_3_ particles adsorbed onto SiO_2_ increased, the positive zeta potential of the surface also increased which generated a shielding effect preventing the subsequent Al_2_O_3_ to be adsorbed onto SiO_2_ and causes the deposition delay. The homogenous distribution of positively charged Al_2_O_3_ particles across the surface of SiO_2_ at almost the same distance between each Al_2_O_3_ particle resulted in the generation of the steric effect that is thought to be equivalent to that of the negatively charged SiO_2_ particle. Hence, this results in the achievement of iso-electric point. In the work reported by Xu et al., they have reported an almost similar observation where, by controlling the charge density of polyelectrolyte, the membrane film roughness and distance variation from the substrate as well as the importance of steric restrictions to the ion to ion spacing in polyelectrolyte pairing were shown [30].

**Fig. 7:**
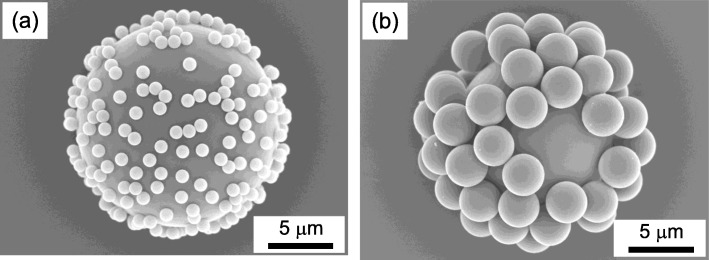
SEM images of the SiO_2_ composite particles that consist of **a** 1-μm SiO_2_ particles and **b** 4-μm SiO_2_ particles decorated on a 16-μm SiO_2_ particle by EA method which demonstrated homogenous distribution

**Fig. 8:**
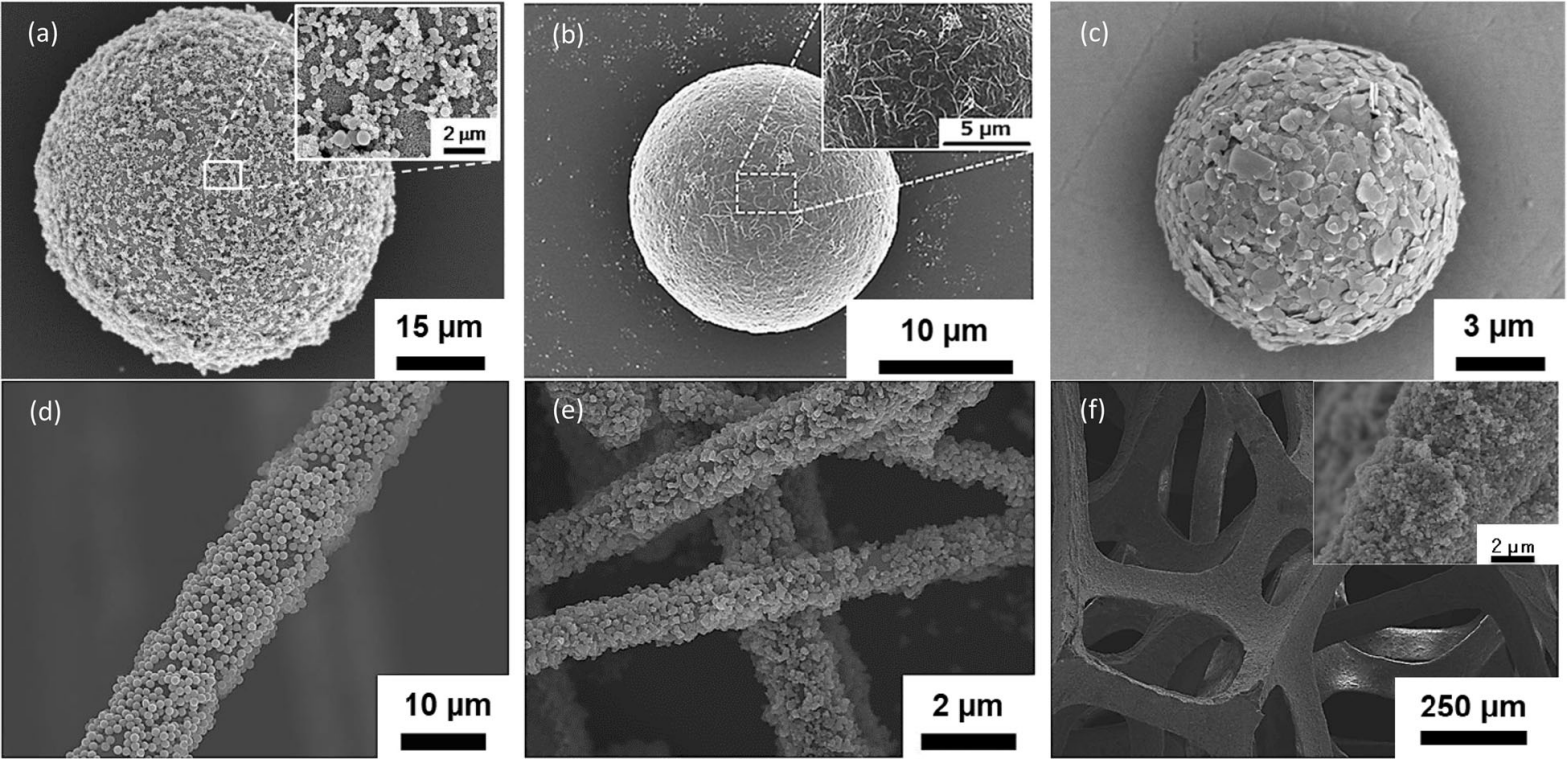
SEM images of the nano and micro-assembled composites obtained by EA method. **a** carbon microspheres-Al_2_O_3_, **b** CNT-PMMA, **c** BN-SiO_2_, **d** SiO_2_-carbon fiber, **e** Al_2_O_3_-SiC whisker, and **f** Al_2_O_3_-urethane foam

In the demonstration of composite particles formation that consist of the same material but of different sizes, it is also important to adjust the surface zeta potential by adjusting the number of alternating polyelectrolyte coatings. Additional file 1: Figure S1 shows the gradual increase of zeta potential with the number of alternating PDDA and PSS coatings. The zeta potential increased one fold from approximately − 30 to − 60 mV after four layers of coating. The SEM images of the SiO_2_ composite particles are shown in Fig. 7, which show an excellent distribution of sub-micro SiO_2_ particles on a 16-μm SiO_2_ particle. In Fig. 7a, sub-microsized SiO_2_ particles of approximately 1 μm are seen homogenously distributed across the surface of a 16 μm SiO_2_ particle while in Fig. 7b, larger SiO_2_ microparticles of approximately 4 μm are seen to be well distributed in a similar fashion. This shows that by adjusting the strength of the surface charge, larger additive particles could be also utilized for the fabrication of composite materials via the EA method. To further demonstrate the feasibility and applicability of this novel method, various materials such as Al_2_O_3_, PMMA, CNT, BN, carbon fiber, SiC, and urethane involving various forms such as fiber as well as whisker and irregular-structured foam were used for the formation of composite. The morphologies of the composites obtained are shown in Fig. 8 indicating homogenous decoration of desired additive particles onto various primary particles and frameworks via the EA method. In Fig. 8a–c, the decoration of different structures such as carbon microspheres, high aspect ratio CNT, and BN nanosheets on different materials that consist of Al_2_O_3_, PMMA, and SiO_2_ microsphere are shown respectively. On the other hand, the homogenous decoration of SiO_2_ and Al_2_O_3_ nanoparticles on non-spherical and irregular structures was demonstrated on carbon fiber, SiC whisker, and urethane foam, as shown in Fig. 8d–f respectively. Therefore, this unique work has demonstrated a novel technique of controlled micro- and nano-assembly that has a huge potential for material design covering various materials as well as morphological dimensions which could offer influential impact toward the development and design of composite materials for precision manufacturing technologies. The limitations of EA method are prerequisite aqueous preparation of material that has a density higher than water (1 g/cm^3^), the difficulty in large scale production, and the requirement of multiple times cleansing in order to remove the excess polyelectrolytes from the solution. However, from this project, an advanced large-scale precursors’ surface charge-modifying system has been developed for a scale-up production of advanced material nano-assembly using a customized equipment with real-time monitoring. This equipment enables the control and alteration of surface charge zeta potential (positive/negative) of a large volume aqueous solution (approximately 10 l) containing the designated starting precursor materials. After achieving the desired zeta potential, the surface-charged modified starting materials are mixed to promote the subsequent electrostatic adsorption to generate the desired composite material.

Using the EA method reported in this work, various composite materials have been fabricated and reported by our group for applications such as controlled mechanical properties of alumina-based carbon composite [24], rapid room temperature formation of composite ceramic films by aerosol deposition [25], controlled IR light-shielding property of PMMA-ITO polymer composite [21], and rechargeable Fe-air battery [20]. In a recent work involving the fabrication of PMMA polymer matrix composite with ITO nanoparticles, PMMA-ITO composite pellet that demonstrated a good transparency in the visible light region while allowing the control of IR light-shielding effect by controlling the amount of ITO nanoparticle incorporation has been reported [21]. Therefore, besides inorganic materials, this shows that the electrostatic assembly method can be applied for polymeric materials as well.

## Conclusions

The feasibility to control the additive particle’s coverage on a primary particle in a composite fabrication is demonstrated in this novel work. The fundamental experimental work was carried out by decoration of Al_2_O_3_ nanoparticles on SiO_2_ microparticles as a function of surface coverage and reaction time. Control decoration with surface coverages of 25, 50, and 75% were also demonstrated by adjusting the amount of additive and EA time. Toward the advancement of nanoscale material design, we have also demonstrated the feasibility to achieve micro- and nano-assembly of particle composites on a wide range of materials with various morphological structures at room temperature using an EA method. A superior homogeneity with controllable surface coverage is also demonstrated in this novel work. The possible applications of the fabricated composite materials using the EA method are selective laser sintering, aerosol deposition of composite ceramic films, IR-shielding materials, and rechargeable metal-air battery. The systematic findings of this work could lay a platform for nanoscale material design toward more sophisticated nanofabrication in the future.

## Additional Files

Additional file 1: **Figure S1.** The zeta potential increased with number of alternating PDDA and PSS coatings. (DOCX 36 kb)

## Data Availability

All data generated or analyzed during this study are included in this published article [and its supplementary information files].

## Abbreviations

BN: Boron nitride
CNT: Carbon nanotube
EA: Electrostatic adsorption
PDDA: Polydiallyldimethylammonium chloride
PMMA: Polymethyl methacrylate
PSS: Polysodium styrenesulfonate
SEM: Scanning electron microscope
SiC: Silicon carbide
