# Supplementary material for: Micro- and Nano-assembly of Composite Particles by Electrostatic Adsorption
Source: Nanoscale Res Lett. 2019 Aug 28;14:297. doi: 10.1186/s11671-019-3129-1 (PMC6713769; doi:10.1186/s11671-019-3129-1)
Supplement: Supplementary file 1 — Figure S1. The zeta potential increased with number of alternating PDDA and PSS coatings. (DOCX 36 kb) [file 11671_2019_3129_MOESM1_ESM.docx]

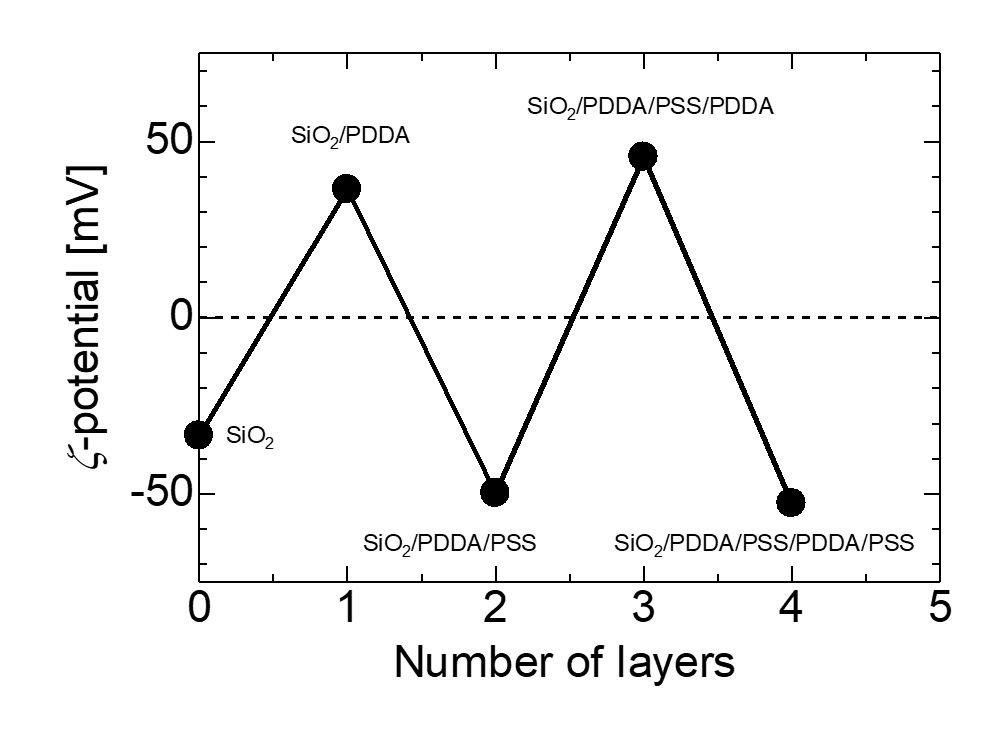


Fig. S1: The surface charge of SiO_2_ particles was controlled and modified by inducing multiple alternating PDDA and PSS polyelectrolyte coatings. The zeta potential obtained shows that with a higher number of alternating polyelectrolyte coating, a higher and more stable zeta potential is obtained for the subsequent electrostatic assembly process.
